# Supplementary material for: Demographic characteristics, clinical symptoms, biochemical markers and probability of occurrence of severe dengue: A multicenter hospital-based study in Bangladesh
Source: PLoS Negl Trop Dis. 2023 Mar 15;17(3):e0011161. doi: 10.1371/journal.pntd.0011161 (PMC10042364; doi:10.1371/journal.pntd.0011161)
Supplement: S1 Questionnaire — (DOCX) [file pntd.0011161.s002.docx]

**S1 Questionnaire (English): Data collection form**

Please check this box if you/participant has given his/her permission: [ ]

Name of the hospital.

**General Information**

1. Name of the patient:
2. Age: years
3. Sex: Male/ Female
4. Mobile number:
5. Present address (complete):
   1. House
   2. Road
   3. Ward/locality
   4. City/District
6. Blood group:
7. Educational qualification:
   1. Children (pre-school)
   2. Illiterate
   3. Primary
   4. SSC
   5. Graduate
8. Monthly income of family (BDT):
   1. <15000
   2. 15000 - <25000
   3. 25000 - <50000
   4. 50000 - <100000
   5. 100000 and above
9. Type of residence
   1. Flat; If yes, which story:
   2. Single storied house
   3. Tin shade house
   4. Slum
   5. Others
10. Occupation:
    1. Service
    2. Business
    3. Doctor/Nurse/Healthworker
    4. Laborer
    5. Student
    6. Children (pre-school)
    7. Others
11. Where were you within 10 days before onset of fever
    1. Dhaka
    2. Outside Dhaka (Which District? )
12. Did you ever suffer from Dengue or Chikungunya?
    1. Dengue
    2. Chikungunya
    3. None
13. Do have any problem listed below (Co-morbidities)
    1. Hypertension
    2. Diabetes
    3. Stroke
    4. Heart Disease
    5. Chronic kidney disease
    6. COPD
    7. Others:
14. Laboratory test results for dengue
    1. NS1: Positive/ Negative/ Not done
    2. IgM: Positive/ Negative/ Not done
    3. IgG: Positive/ Negative/ Not done

**Fever related information**

1. When the did the fever start? Date (dd/mm/yyyy):
2. For how many days you are hospitalized? Days
3. What was the highest recorded temperature?
   1. 100
   2. 101
   3. 102
   4. 103
   5. 104
   6. 105
   7. Not measured
   8. Can’t remember
4. What was the duration of fever? Days
5. Were there any of the following symptoms associated with fever?
   1. Headache: 🞎 Yes 🞎 No 🞎 Can’t remember
   2. Retro-orbital pain: 🞎 Yes 🞎 No 🞎 Can’t remember
   3. Back pain: 🞎 Yes 🞎 No 🞎 Can’t remember
   4. Muscle pain: 🞎 Yes 🞎 No 🞎 Can’t remember
   5. Chills and/or rigor: 🞎 Yes 🞎 No 🞎 Can’t remember
   6. Runny nose, cough or soar throat, malaise? 🞎 Yes 🞎 No 🞎 Can’t remember
   7. Lethargy: 🞎 Yes 🞎 No 🞎 Can’t remember

**Skin manifestations (After start of dengue symptoms)**

1. Was there any rash in body? 🞎 Yes 🞎 No
2. Was there itching? 🞎 Yes 🞎 No
3. Was/is there any hair loss? 🞎 Yes 🞎 No
4. Was there any black spot in the body? 🞎 Yes 🞎 No
5. Was there any wound? 🞎 Yes 🞎 No
6. Was there oral ulcer? 🞎 Yes 🞎 No
7. Was there any pustule in body? 🞎 Yes 🞎 No

**Arthralgia/Arthritis related manifestations (After start of dengue symptoms)**

1. Was there any joint pain during fever? 🞎 Yes 🞎 No
   1. If yes, Pain rating: 1/2/3/4/5/6/7/8/9/10
2. When the did the join pain start? 🞎 Before fever 🞎 After fever
3. In which joints pain was present?
   1. Finger
   2. Wrist
   3. Lower back
   4. Knee
   5. Ankle
   6. Foot
   7. Shoulder
   8. Chest
   9. Others:
4. When did the joint pain occur? 🞎 All times 🞎 Sometime
5. Was there any joint swelling? 🞎 Yes 🞎 No 🞎 Can’t remember
6. If yes, which joint?
   1. Finger (hand)
   2. Hand joints
   3. Foot joints
   4. Finger (foot)
   5. Others:
7. Was there redness of joint? 🞎 Yes 🞎 No 🞎 Can’t remember
8. Was joint pain involved both side and similar joints? 🞎 Yes 🞎 No 🞎 Can’t remember
9. For how many days joint pain was present? Days
10. Is there any joint stiffness at morning? 🞎 Yes 🞎 No

**Danger signs (After start of dengue symptoms)**

Regarding hemorrhage:

1. Was there any hemorrhage with fever? 🞎 Yes 🞎 No
   1. If yes, when? 🞎 With fever 🞎 After fever
2. After how many days of fever hemorrhage started? Days
3. From which sites hemorrhage occurred?
   1. Gum
   2. Nose
   3. Under the skin
   4. With stool: Fresh red blood/ tar like black blood
   5. With cough (hemoptysis)
   6. With vomit (hematemesis)
   7. Excess bleeding during menstruation
   8. Conjunctiva
   9. With urine (hematuria)
4. Was platelet reduced? 🞎 Yes 🞎 No
5. What was the minimum platelet count? (Verify from medical report)
6. Was there any need to take blood transfusion? 🞎 Yes 🞎 No
   1. If yes, how many bags of
      1. Platelet concentrate: ………………(total) bags
      2. Whole blood: …………………(total) bags
      3. Plasma: ………………(Total) bags

**Gastrointestinal manifestations (After start of dengue symptoms)**

1. Did you vomit? 🞎 Yes 🞎 No
2. Did you have loose motion? 🞎 Yes 🞎 No
3. Was there abdominal pain? 🞎 Yes 🞎 No
4. Was there decreased appetite? 🞎 Yes 🞎 No
5. Was there constipation? 🞎 Yes 🞎 No

**Signs of Plasma Leakage (After start of dengue symptoms)**

1. Was there any sign of edema/plasma leakage? 🞎 Yes 🞎 No
   1. If yes, where?
      1. Abdomen (ascites)
      2. Chest (pleural effusion)
      3. Legs (pedal edema)
      4. Whole body (anasarca)
2. Did the patient’s blood pressure decrease? 🞎 Yes 🞎 No 🞎 Can’t remember
3. Did the patient’s body become cold? 🞎 Yes 🞎 No 🞎 Can’t remember
4. Was there excessive sweating? 🞎 Yes 🞎 No 🞎 Can’t remember
5. Was there any decrease in or cessation of urinary output? 🞎 Yes 🞎 No 🞎 Can’t remember
6. Was there any damage of more than one organ? 🞎 Yes 🞎 No
   1. If yes, which organs?
      1. Liver
      2. Kidney
      3. Heart
      4. Lungs
      5. Brain
7. Did the patient loose consciousness? 🞎 Yes 🞎 No

**Other symptoms (After start of dengue symptoms)**

1. Was there blurring of vision? 🞎 Yes 🞎 No 🞎 Can’t remember
2. Did the patient talk irrelevant? 🞎 Yes 🞎 No 🞎 Can’t remember
3. Did the patient have difficulty in identifying familiar persons? 🞎 Yes 🞎 No 🞎 Can’t remember
4. Did the patient see or hear unnatural things after fever? 🞎 Yes 🞎 No 🞎 Can’t remember
5. Did the patient lose his/her memory? 🞎 Yes 🞎 No 🞎 Can’t remember
6. Did the patients suffer altered mental state? 🞎 Yes 🞎 No 🞎 Can’t remember
7. Did the patient’s neck become stiff? 🞎 Yes 🞎 No 🞎 Can’t remember
8. Did the patients suffer convulsion? 🞎 Yes 🞎 No 🞎 Can’t remember
9. Did the patient’s mouth become deviated? 🞎 Yes 🞎 No 🞎 Can’t remember
10. Was there any loss of sensation of hands and feet? 🞎 Yes 🞎 No 🞎 Can’t remember
11. Was there any chest tightness? 🞎 Yes 🞎 No 🞎 Can’t remember
12. Was there any palpitation? 🞎 Yes 🞎 No 🞎 Can’t remember
13. Was there any difficulty in respiration? 🞎 Yes 🞎 No 🞎 Can’t remember
14. Was there any bluish discoloration of tongue or fingers of hands and feet? 🞎 Yes 🞎 No 🞎 Can’t remember

**Important signs**

1. Anemia: 🞎 Yes 🞎 No
2. Jaundice: 🞎 Yes 🞎 No
3. Cyanosis: 🞎 Yes 🞎 No
4. Dehydration: 🞎 Yes 🞎 No
5. Palpable lymph nodes: 🞎 Yes 🞎 No
6. Hepatomegaly: 🞎 Yes 🞎 No; (if palpable), size:
7. spleenomegaly: 🞎 Yes 🞎 No; (if palpable), size:

**Important Investigations**

**Hematological parameters**

a. HCT (%): 􀆱 Raised (> 20% from baseline)  Reduced 􀆱 Within normal

b. Hemoglobin: 􀆱 Reduced (Lowest value: ……………….... ) 􀆱 Within normal

c. Platelet count: 􀆱 Reduced (Lowest value: ……………………….) 􀆱 Within normal

d. WBC count: 􀆱 Reduced (Lowest value: …………………………). 􀆱 Within normal

**2. Liver function tests**

a. Serum bilirubin: 􀆱 Raised (Highest value: …………...) 􀆱 Within normal 􀆱 Not done

b. ALT (SGOT): 􀆱 Raised (Highest value: ……………...) Within normal 􀆱 Not done

c. AST (SGPT):  􀆱 Raised (Highest value: ...…………...) 􀆱 Within normal 􀆱 Not done

d. Serum albumin. 􀆱 Reduced 􀆱 Within normal 􀆱 Not done

**3. Coagulation profile**

1. PT: 􀆱 Prolonged (>3s from control) 􀆱 Within normal 􀆱 Not done
2. APTT: 􀆱 Prolonged  􀆱 Within normal 􀆱 Not done

**4. Other**

a. Serum creatinine: 􀆱 Increased 􀆱 Within normal 􀆱 Not done

b. Blood sugar: 􀆱 Reduced 􀆱 Increased 􀆱 Within normal 􀆱 Not done

c. Urine R/M/E (Albuminuria): 􀆱 Present 􀆱 Absent 􀆱 Not Done

d. Electrolyte: 􀆱 Done 􀆱 Not Done; if done,

- Na: 􀆱 Reduced 􀆱 Increased 􀆱 Normal
- K: 􀆱 Reduced 􀆱 Increased 􀆱 Normal

**5. Echocardiography**

􀆱 Abnormal (Specify: ……………………………………………………………) 􀆱 Within normal 􀆱 Not done

**6. ECG**

􀆱 Abnormal (Specify: ………………………………………………………………….) 􀆱 Within normal 􀆱 Not done

**Last diagnosis at hospital**

1. What was the last diagnosis at hospital? 🞎 Dengue Fever 🞎 Dengue hemorrhagic fever without shock 🞎 Dengue hemorrhagic fever with shock or Dengue shock syndrome 🞎 Expanded Dengue Syndrome

New classification of cases (according to Dengue Guideline, Bangladesh)

🞎 Group A     🞎 Group B       🞎 Group C

Any other symptoms/signs/ Note:……………………………………………………………………………

……………………………………………………………………………………………………………….

……………………………………………………………………………………………………………….

Name of the data collector: Date (dd/mm/yyyy)….
