# Supplementary figures and images for: Demographic characteristics, clinical symptoms, biochemical markers and probability of occurrence of severe dengue: A multicenter hospital-based study in Bangladesh
Source: PLoS Negl Trop Dis. 2023 Mar 15;17(3):e0011161. doi: 10.1371/journal.pntd.0011161 (PMC10042364; doi:10.1371/journal.pntd.0011161)

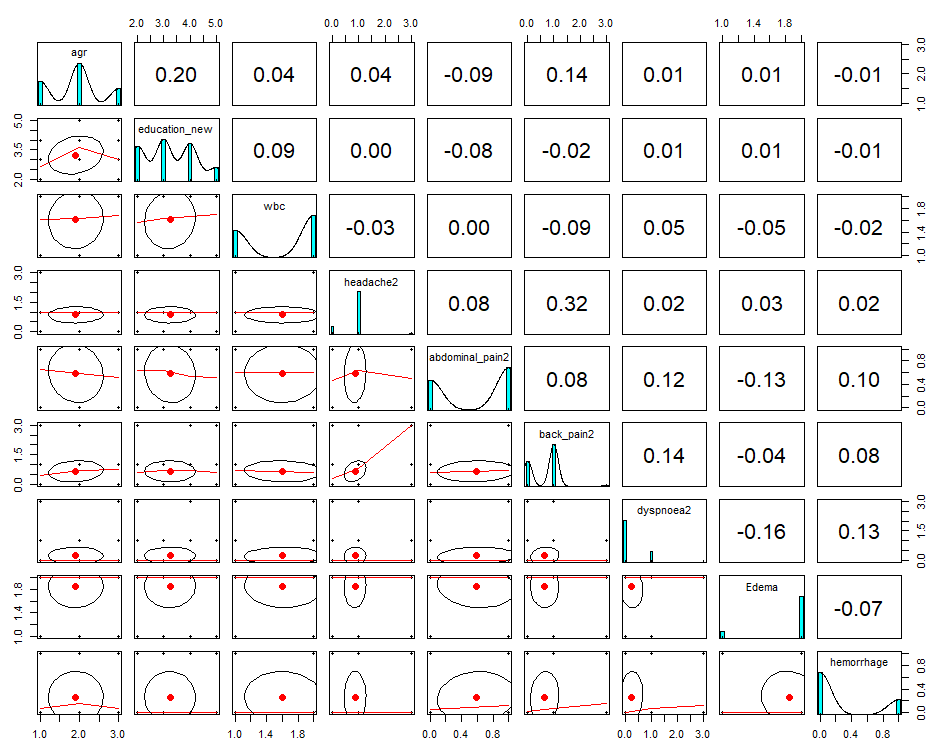

Supplement: S2 Fig — (TIF) [file pntd.0011161.s004.tif]

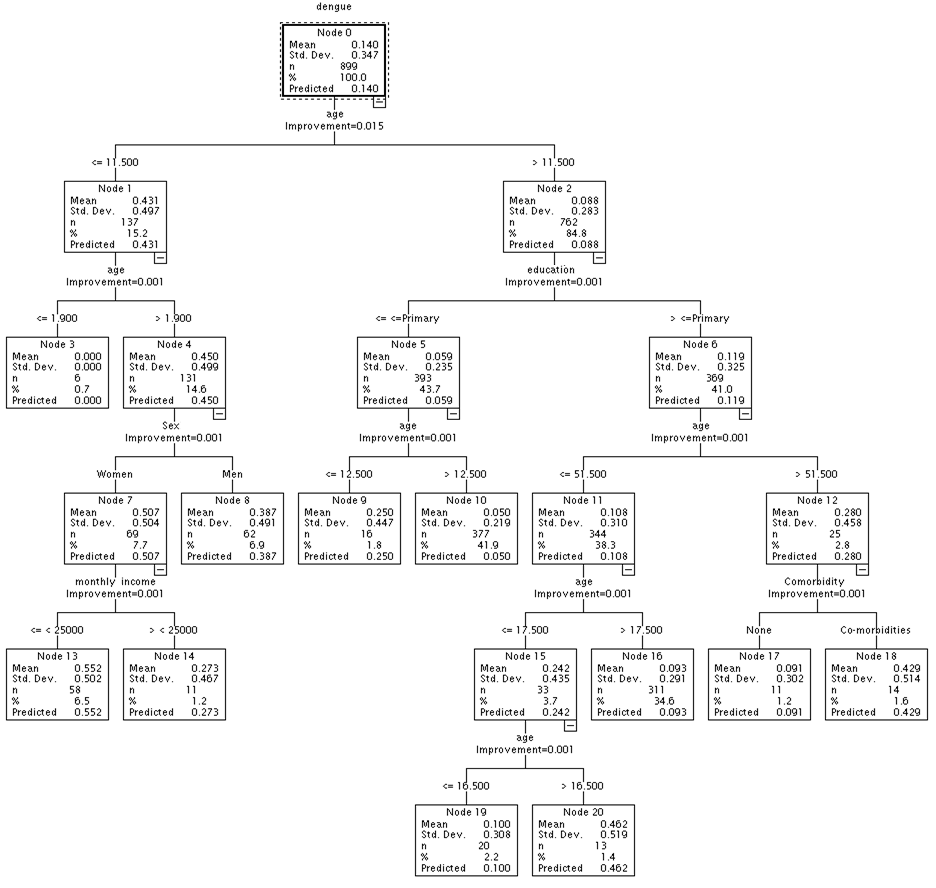

Supplement: S3 Fig — (TIF) [file pntd.0011161.s005.tif]

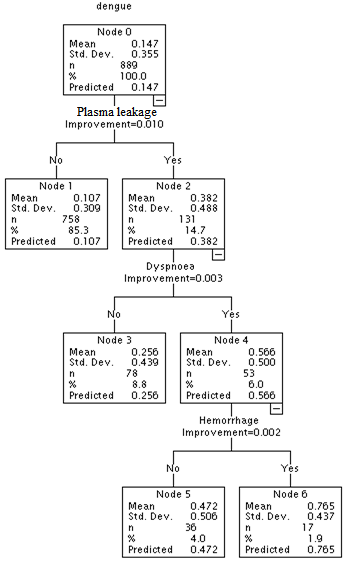

Supplement: S4 Fig — (TIF) [file pntd.0011161.s006.tif]

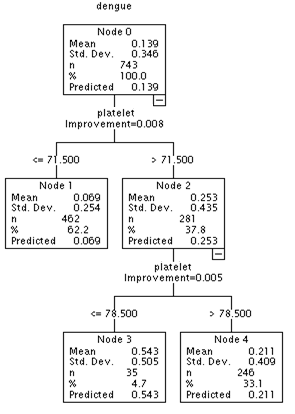

Supplement: S5 Fig — (TIF) [file pntd.0011161.s007.tif]
