# Supplementary material for: Demographic characteristics, clinical symptoms, biochemical markers and probability of occurrence of severe dengue: A multicenter hospital-based study in Bangladesh
Source: PLoS Negl Trop Dis. 2023 Mar 15;17(3):e0011161. doi: 10.1371/journal.pntd.0011161 (PMC10042364; doi:10.1371/journal.pntd.0011161)
Supplement: S1 Table — * Values are presented as n (%). (DOCX) [file pntd.0011161.s008.docx]

**S1 Table. Clinical features of the patients grouped by severity of dengue** ^*^**.**

|  | **Overall** | **Non-severe dengue** | **Severe dengue** | **P-value** |
| --- | --- | --- | --- | --- |
|  | **(N=1090)** | **(N=932)** | **(N=158)** |  |
| **Fever** |  |  |  | 0.09 |
| Yes | 1034 (94.9) | 889 (95.4) | 145 (91.8) |  |
| No | 56 (5.1) | 43 (4.6) | 13 (8.2) |  |
| **Muscle pain** |  |  |  | 0.84 |
| Yes | 691 (63.4) | 594 (63.7) | 97 (61.4) |  |
| No | 367 (33.7) | 313 (33.6) | 54 (34.2) |  |
| Missing | 32 (2.9) | 25 (2.7) | 7 (4.4) |  |
| **Vomiting** |  |  |  | 0.15 |
| Yes | 837 (76.8) | 707 (75.9) | 130 (82.3) |  |
| No | 243 (22.3) | 215 (23.1) | 28 (17.7) |  |
| Missing | 10 (0.9) | 10 (1.1) | 0 (0) |  |
| **Headache** |  |  |  | 0.01 |
| Yes | 901 (82.7) | 782 (83.9) | 119 (75.3) |  |
| No | 171 (15.7) | 135 (14.5) | 36 (22.8) |  |
| Missing | 18 (1.7) | 15 (1.6) | 3 (1.9) |  |
| **Decreased appetite** |  |  |  | 0.94 |
| Yes | 869 (79.7) | 744 (79.8) | 125 (79.1) |  |
| No | 215 (19.7) | 183 (19.6) | 32 (20.3) |  |
| Missing | 6 (0.6) | 5 (0.5) | 1 (0.6) |  |
| **Abdominal pain** |  |  |  | 0.04 |
| Yes | 631 (57.9) | 527 (56.5) | 104 (65.8) |  |
| No | 450 (41.3) | 397 (42.6) | 53 (33.5) |  |
| Missing | 9 (0.8) | 8 (0.9) | 1 (0.6) |  |
| **Rash** |  |  |  | 0.85 |
| Yes | 277 (25.4) | 235 (25.2) | 42 (26.6) |  |
| No | 795 (72.9) | 680 (73.0) | 115 (72.8) |  |
| Missing | 18 (1.7) | 17 (1.8) | 1 (0.6) |  |
| **Cough** |  |  |  | 0.79 |
| Yes | 380 (34.9) | 322 (34.5) | 58 (36.7) |  |
| No | 644 (59.1) | 551 (59.1) | 93 (58.9) |  |
| Missing | 66 (6.1) | 59 (6.3) | 7 (4.4) |  |
| **Back pain** |  |  |  | 0.02 |
| Yes | 651 (59.7) | 571 (61.3) | 80 (50.6) |  |
| No | 389 (35.7) | 320 (34.3) | 69 (43.7) |  |
| Missing | 50 (4.6) | 41 (4.4) | 9 (5.7) |  |
| **Joint pain** |  |  |  | 0.75 |
| Yes | 516 (47.3) | 440 (47.2) | 76 (48.1) |  |
| No | 556 (51.0) | 479 (51.4) | 77 (48.7) |  |
| Missing | 18 (1.7) | 13 (1.4) | 5 (3.2) |  |
| **Dehydration** |  |  |  | 0.54 |
| Yes | 227 (20.8) | 192 (20.6) | 35 (22.2) |  |
| No | 820 (75.2) | 709 (76.1) | 111 (70.3) |  |
| Missing | 43 (3.9) | 31 (3.3) | 12 (7.6) |  |
| **Dyspnoea** |  |  |  | <0.01 |
| Yes | 223 (20.5) | 163 (17.5) | 60 (38.0) |  |
| No | 847 (77.7) | 752 (80.7) | 95 (60.1) |  |
| Missing | 20 (1.8) | 17 (1.8) | 3 (1.9) |  |
| **Itchiness** |  |  |  | 1.00 |
| Yes | 330 (30.3) | 282 (30.3) | 48 (30.4) |  |
| No | 738 (67.7) | 629 (67.5) | 109 (69.0) |  |
| Missing | 22 (2.0) | 21 (2.3) | 1 (0.6) |  |
| **Lethargy** |  |  |  | 0.17 |
| Yes | 873 (80.1) | 751 (80.6) | 122 (77.2) |  |
| No | 162 (14.9) | 132 (14.2) | 30 (19.0) |  |
| Missing | 55 (5.0) | 49 (5.3) | 6 (3.8) |  |
| **Plasma leakage** |  |  |  | <0.01 |
| Yes | 159 (14.6) | 106 (11.4) | 53 (33.5) |  |
| No | 887 (81.4) | 789 (84.7) | 98 (62.0) |  |
| Missing | 44 (4.0) | 37 (4.0) | 7 (4.4) |  |
| **Hemorrhage** |  |  |  | <0.01 |
| Yes | 272 (25.0) | 216 (23.2) | 56 (35.4) |  |
| No | 766 (70.3) | 682 (73.2) | 84 (53.2) |  |
| Missing | 52 (4.8) | 34 (3.6) | 18 (11.4) |  |

^*^ Values are presented as n (%).
