# Supplementary material for: Demographic characteristics, clinical symptoms, biochemical markers and probability of occurrence of severe dengue: A multicenter hospital-based study in Bangladesh
Source: PLoS Negl Trop Dis. 2023 Mar 15;17(3):e0011161. doi: 10.1371/journal.pntd.0011161 (PMC10042364; doi:10.1371/journal.pntd.0011161)
Supplement: S2 Table — Abbreviation: WBC, white blood cell; ALT, alanine transaminase; AST, aspartate transaminase. * Values are presented as n (%). (DOCX) [file pntd.0011161.s009.docx]

## **S2 Table**. **Laboratory findings of the patients grouped by severity of dengue** ^*^**.**

|  | **Overall** | **Non-severe** | **Severe** | **P-value** |
| --- | --- | --- | --- | --- |
|  | **(N=1090)** | **(N=932)** | **(N=158)** |  |
| **Platelet, 10^9^/L** |  |  |  | 0.14 |
| Reduced | 972 (89.2) | 840 (90.1) | 132 (83.5) |  |
| Within normal | 103 (9.4) | 83 (8.9) | 20 (12.7) |  |
| Missing | 15 (1.4%) | 9 (1.0%) | 6 (3.8%) |  |
| **Hemoglobin, g/dL** | |  |  | 0.17 |
| Reduced | 315 (28.9) | 278 (29.8) | 37 (23.4) |  |
| Within normal | 757 (69.4) | 642 (68.9) | 115 (72.8) |  |
| Missing | 18 (1.7%) | 12 (1.3%) | 6 (3.8%) |  |
| **WBC, 10^9^/L** |  |  |  | 0.03 |
| Reduced | 404 (37.1) | 363 (38.9) | 41 (25.9) |  |
| Within normal | 624 (57.2) | 518 (55.6) | 106 (67.1) |  |
| Missing | 62 (5.7%) | 51 (5.5%) | 11 (7.0%) |  |
| **ALT, U/L** |  |  |  | 0.16 |
| Raised | 183 (16.8) | 166 (17.8) | 17 (10.8) |  |
| Within normal | 99 (9.1) | 84 (9.0) | 15 (9.5) |  |
| Not done | 700 (64.2) | 598 (64.2) | 102 (64.6) |  |
| Missing | 108 (9.9%) | 84 (9.0%) | 24 (15.2%) |  |
| **AST, U/L** |  |  |  | 0.19 |
| Raised | 148 (13.6) | 135 (14.5) | 13 (8.2) |  |
| Within normal | 87 (8.0) | 73 (7.8) | 14 (8.9) |  |
| Not done | 711 (65.2) | 614 (65.9) | 97 (61.4) |  |
| Missing | 144 (13.2%) | 110 (11.8%) | 34 (21.5%) |  |

WBC, white blood cell; ALT, alanine transaminase; AST, aspartate transaminase.

^*^ Values are presented as n (%).
