# Supplementary material for: Demographic characteristics, clinical symptoms, biochemical markers and probability of occurrence of severe dengue: A multicenter hospital-based study in Bangladesh
Source: PLoS Negl Trop Dis. 2023 Mar 15;17(3):e0011161. doi: 10.1371/journal.pntd.0011161 (PMC10042364; doi:10.1371/journal.pntd.0011161)
Supplement: S3 Table — Abbreviation: WBC, white blood cell; ALT, alanine transaminase; AST, aspartate transaminase. (DOCX) [file pntd.0011161.s010.docx]

## **S3 Table**. **Association of demographic characteristics, clinical symptoms, and biochemical markers with the risk of severe dengue in crude logistic regression model.**

|  | B | S.E. | Wald | P value | OR | 95% CI for OR | |
| --- | --- | --- | --- | --- | --- | --- | --- |
|  |  |  |  |  |  | Lower | Upper |
| **Age group (years)** |  |  |  |  |  |  |  |
| <18 | 0.00 (Ref.) | 0.00 | - | 1.00 | 1.00 (Ref.) | 1.00 | 1.00 |
| 18-39 | -1.50 | 0.20 | 57.77 | 0.00 | 0.22 | 0.15 | 0.33 |
| 40 and above | -1.40 | 0.27 | 27.83 | 0.00 | 0.25 | 0.15 | 0.42 |
| **Sex** |  |  |  |  |  |  |  |
| Men | 0.00 (Ref.) | 0.00 | - | 1.00 | 1.00 (Ref.) | 1.00 | 1.00 |
| Women | 0.32 | 0.17 | 3.38 | 0.07 | 1.37 | 0.98 | 1.93 |
| **Education** |  |  |  |  |  |  |  |
| Illiterate or Children | 0.00 (Ref.) | 0.00 | - | 1.00 | 1.00 (Ref.) | 1.00 | 1.00 |
| Primary | -0.83 | 0.23 | 13.38 | 0.00 | 0.43 | 0.28 | 0.68 |
| Secondary and higher Secondary School | -0.54 | 0.22 | 6.06 | 0.01 | 0.58 | 0.38 | 0.90 |
| Graduate | -0.73 | 0.33 | 4.99 | 0.03 | 0.48 | 0.25 | 0.91 |
| **Monthly income (BDT)** |  |  |  |  |  |  |  |
| < 15000 | 0.00 (Ref.) | 0.00 | - | 1.00 | 1.00 (Ref.) | 1.00 | 1.00 |
| 15000-25000 | -0.22 | 0.21 | 1.03 | 0.31 | 0.81 | 0.53 | 1.22 |
| 25000-50000 | 0.20 | 0.24 | 0.72 | 0.40 | 1.22 | 0.77 | 1.94 |
| 50000 and above | 0.02 | 0.36 | 0.00 | 0.95 | 1.02 | 0.50 | 2.07 |
| **Type of residence** |  |  |  |  |  |  |  |
| Flat | 0.00 (Ref.) | 0.00 | - | 1.00 | 1.00 (Ref.) | 1.00 | 1.00 |
| Single storied house | -0.02 | 0.28 | 0.00 | 0.95 | 0.98 | 0.56 | 1.71 |
| Tin shade house and slum | 0.02 | 0.19 | 0.01 | 0.93 | 1.02 | 0.70 | 1.47 |
| **History of comorbidity** |  |  |  |  |  |  |  |
| No | 0.00 (Ref.) | 0.00 | - | 1.00 | 1.00 (Ref.) | 1.00 | 1.00 |
| Yes | 0.10 | 0.23 | 0.20 | 0.65 | 1.11 | 0.71 | 1.74 |
| **History of dengue** |  |  |  |  |  |  |  |
| None | 0.00 (Ref.) | 0.00 | - | 1.00 | 1.00 (Ref.) | 1.00 | 1.00 |
| Dengue | -0.22 | 0.62 | 0.12 | 0.73 | 0.81 | 0.24 | 2.74 |
| Chikungunya | -0.26 | 0.30 | 0.73 | 0.39 | 0.77 | 0.43 | 1.39 |
| **Fever** |  |  |  |  |  |  |  |
| No | 0.00 (Ref.) | 0.00 | - | 1.00 | 1.00 (Ref.) | 1.00 | 1.00 |
| Yes | -0.62 | 0.33 | 3.52 | 0.06 | 0.54 | 0.28 | 1.03 |
| **Muscle pain** |  |  |  |  |  |  |  |
| No | 0.00 (Ref.) | 0.00 | - | 1.00 | 1.00 (Ref.) | 1.00 | 1.00 |
| Yes | -0.05 | 0.18 | 0.09 | 0.76 | 0.95 | 0.66 | 1.36 |
| **Headache** |  |  |  |  |  |  |  |
| No | 0.00 (Ref.) | 0.00 | - | 1.00 | 1.00 (Ref.) | 1.00 | 1.00 |
| Yes | -0.56 | 0.21 | 7.01 | 0.01 | 0.57 | 0.38 | 0.86 |
| **Vomiting** |  |  |  |  |  |  |  |
| No | 0.00 (Ref.) | 0.00 | - | 1.00 | 1.00 (Ref.) | 1.00 | 1.00 |
| Yes | 0.34 | 0.22 | 2.41 | 0.12 | 1.41 | 0.91 | 2.18 |
| **Decreased appetite** |  |  |  |  |  |  |  |
| No | 0.00 (Ref.) | 0.00 | - | 1.00 | 1.00 (Ref.) | 1.00 | 1.00 |
| Yes | -0.04 | 0.21 | 0.03 | 0.85 | 0.96 | 0.63 | 1.46 |
| **Abdominal pain** |  |  |  |  |  |  |  |
| No | 0.00 (Ref.) | 0.00 | - | 1.00 | 1.00 (Ref.) | 1.00 | 1.00 |
| Yes | 0.39 | 0.18 | 4.64 | 0.03 | 1.48 | 1.04 | 2.11 |
| **Rash** |  |  |  |  |  |  |  |
| No | 0.00 (Ref.) | 0.00 | - | 1.00 | 1.00 (Ref.) | 1.00 | 1.00 |
| Yes | 0.06 | 0.20 | 0.08 | 0.78 | 1.06 | 0.72 | 1.55 |
| **Cough** |  |  |  |  |  |  |  |
| No | 0.00 (Ref.) | 0.00 | - | 1.00 | 1.00 (Ref.) | 1.00 | 1.00 |
| Yes | 0.07 | 0.18 | 0.13 | 0.72 | 1.07 | 0.75 | 1.52 |
| **Back pain** |  |  |  |  |  |  |  |
| No | 0.00 (Ref.) | 0.00 | - | 1.00 | 1.00 (Ref.) | 1.00 | 1.00 |
| Yes | -0.43 | 0.18 | 5.83 | 0.02 | 0.65 | 0.46 | 0.92 |
| **Joint pain** |  |  |  |  |  |  |  |
| No | 0.00 (Ref.) | 0.00 | - | 1.00 | 1.00 (Ref.) | 1.00 | 1.00 |
| Yes | 0.07 | 0.17 | 0.17 | 0.68 | 1.07 | 0.76 | 1.51 |
| **Dehydration** |  |  |  |  |  |  |  |
| No | 0.00 (Ref.) | 0.00 | - | 1.00 | 1.00 (Ref.) | 1.00 | 1.00 |
| Yes | 0.15 | 0.21 | 0.52 | 0.47 | 1.16 | 0.77 | 1.76 |
| **Dyspnoea** |  |  |  |  |  |  |  |
| No | 0.00 (Ref.) | 0.00 | - | 1.00 | 1.00 (Ref.) | 1.00 | 1.00 |
| Yes | 1.07 | 0.19 | 33.00 | 0.00 | 2.91 | 2.02 | 4.20 |
| **Itchiness** |  |  |  |  |  |  |  |
| No | 0.00 (Ref.) | 0.00 | - | 1.00 | 1.00 (Ref.) | 1.00 | 1.00 |
| Yes | -0.02 | 0.19 | 0.01 | 0.92 | 0.98 | 0.68 | 1.42 |
| **Lethargy** |  |  |  |  |  |  |  |
| No | 0.00 (Ref.) | 0.00 | - | 1.00 | 1.00 (Ref.) | 1.00 | 1.00 |
| Yes | -0.34 | 0.22 | 2.24 | 0.13 | 0.71 | 0.46 | 1.11 |
| **Plasma leakage** |  |  |  |  |  |  |  |
| No | 0.00 (Ref.) | 0.00 | - | 1.00 | 1.00 (Ref.) | 1.00 | 1.00 |
| Yes | 1.39 | 0.20 | 48.76 | 0.00 | 4.03 | 2.72 | 5.95 |
| **Hemorrhage** |  |  |  |  |  |  |  |
| No | 0.00 (Ref.) | 0.00 | - | 1.00 | 1.00 (Ref.) | 1.00 | 1.00 |
| Yes | 0.74 | 0.19 | 15.45 | 0.00 | 2.10 | 1.45 | 3.05 |
| **Platelet, 10^9^/L** |  |  |  |  |  |  |  |
| Within normal | 0.00 (Ref.) | 0.00 | - | 1.00 | 1.00 (Ref.) | 1.00 | 1.00 |
| Reduced | -0.43 | 0.27 | 2.58 | 0.11 | 0.65 | 0.39 | 1.10 |
| **WBC, 10^9^/L** |  |  |  |  |  |  |  |
| Within normal | 0.00 (Ref.) | 0.00 | - | 1.00 | 1.00 (Ref.) | 1.00 | 1.00 |
| Reduced | -0.59 | 0.20 | 9.17 | 0.00 | 0.55 | 0.38 | 0.81 |
| **ALT, U/L** |  |  |  |  |  |  |  |
| Within normal | 0.00 (Ref.) | 0.00 | - | 1.00 | 1.00 (Ref.) | 1.00 | 1.00 |
| Raised | -0.56 | 0.38 | 2.16 | 0.14 | 0.57 | 0.27 | 1.21 |
| **AST, U/L** |  |  |  |  |  |  |  |
| Within normal | 0.00 (Ref.) | 0.00 | - | 1.00 | 1.00 (Ref.) | 1.00 | 1.00 |
| Raised | -0.69 | 0.41 | 2.80 | 0.09 | 0.50 | 0.22 | 1.13 |
| **Hemoglobin, g/dL** | |  |  |  |  |  |  |
| Within normal | 0.00 (Ref.) | 0.00 | - | 1.00 | 1.00 (Ref.) | 1.00 | 1.00 |
| Reduced | -0.3 | 0.20 | 2.16 | 0.14 | 0.74 | 0.50 | 1.10 |

WBC, white blood cell; ALT, alanine transaminase; AST, aspartate transaminase.
