# Supplementary material for: Demographic characteristics, clinical symptoms, biochemical markers and probability of occurrence of severe dengue: A multicenter hospital-based study in Bangladesh
Source: PLoS Negl Trop Dis. 2023 Mar 15;17(3):e0011161. doi: 10.1371/journal.pntd.0011161 (PMC10042364; doi:10.1371/journal.pntd.0011161)
Supplement: S4 Table — (DOCX) [file pntd.0011161.s011.docx]

**S4 Table**. **Collinearity analysis (variance inflation factor, VIFs) in multiple logistic regression model.**

|  | VIF |
| --- | --- |
| Age | 1.63 |
| Education | 1.56 |
| Headache | 3.16 |
| Abdominal pain | 1.10 |
| Backpain | 3.38 |
| Dyspnoea | 1.56 |
| Hemorrhage | 5.89 |
| Plasma leakage | 1.08 |
| White blood cell | 5.92 |
