# Supplementary material for: Demographic characteristics, clinical symptoms, biochemical markers and probability of occurrence of severe dengue: A multicenter hospital-based study in Bangladesh
Source: PLoS Negl Trop Dis. 2023 Mar 15;17(3):e0011161. doi: 10.1371/journal.pntd.0011161 (PMC10042364; doi:10.1371/journal.pntd.0011161)
Supplement: S5 Table — * Values are presented as n (%). (DOCX) [file pntd.0011161.s012.docx]

**S5 Table. Clinical features of the patients grouped by severity of dengue and the site of haemorrhage and plasma leakage** ^*^**.**

|  | **Overall** | **Non-severe dengue** | **Severe dengue** | **P-value** |
| --- | --- | --- | --- | --- |
|  | **(N=1090)** | **(N=932)** | **(N=158)** |  |
| Sites of Hemorrhage |  |  |  |  |
| Gum | 93 (8.5%) | 75 (8.0%) | 18 (11%) | 0.2 |
| Hematochezia | 66 (6.1%) | 46 (4.9%) | 20 (13%) | <0.001 |
| Menorrhagia | 43 (3.9%) | 34 (3.6%) | 9 (5.7%) | 0.2 |
| Conjunctiva | 40 (3.7%) | 29 (3.1%) | 11 (7.0%) | 0.017 |
| Hematemesis | 35 (3.2%) | 25 (2.7%) | 10 (6.3%) | 0.016 |
| Nose | 28 (2.6%) | 20 (2.1%) | 8 (5.1%) | 0.050 |
| Under the skin | 25 (2.3%) | 20 (2.1%) | 5 (3.2%) | 0.4 |
| Hemoptysis | 27 (2.5%) | 18 (1.9%) | 9 (5.7%) | 0.010 |
| Hematuria | 27 (2.5%) | 18 (1.9%) | 9 (5.7%) | 0.010 |
| localization of plasma leakage |  |  |  |  |
| Ascites | 78 (7.2%) | 55 (5.9%) | 23 (15%) | <0.001 |
| Pleural effusion | 50 (4.6%) | 28 (3.0%) | 22 (14%) | <0.001 |
| Anasarca | 24 (2.2%) | 13 (1.4%) | 11 (7.0%) | <0.001 |
| Pedal edema | 17 (1.6%) | 12 (1.3%) | 5 (3.2%) | 0.086 |

^*^ Values are presented as n (%).
