# Supplementary material for: Demographic characteristics, clinical symptoms, biochemical markers and probability of occurrence of severe dengue: A multicenter hospital-based study in Bangladesh
Source: PLoS Negl Trop Dis. 2023 Mar 15;17(3):e0011161. doi: 10.1371/journal.pntd.0011161 (PMC10042364; doi:10.1371/journal.pntd.0011161)
Supplement: S6 Table — Abbreviation: DSS, dengue shock syndrome. * Values are presented as n (%). (DOCX) [file pntd.0011161.s013.docx]

## **S6 Table. Clinical features of the patients grouped by severity of dengue and DSS** ^*^**.**

|  | Non-DSS | |  |  | DSS | |  |
| --- | --- | --- | --- | --- | --- | --- | --- |
|  | Non-severe | Severe | *P* value |  | Non-severe | Severe | *P* value |
| Plasma leakage | |  | <0.01 |  |  |  | 0.09 |
| No | 752 (95.9) | 32 (4.1) |  |  | 9 (13.0) | 60 (87.0) |  |
| Yes | 100 (85.5) | 17 (14.5) |  |  | 1 (2.9) | 34 (97.1) |  |
| Dyspnoea | |  | <0.01 |  |  |  | 0.04 |
| No | 720 (95.7) | 32 (4.3) |  |  | 6 (9.1) | 60 (90.9) |  |
| Yes | 154 (88.0) | 21 (12.0) |  |  | 2 (5.6) | 34 (94.4) |  |
| Hemorrhage | |  | 0.05 |  |  |  | 0.47 |
| No | 656 (96.3) | 25 (3.7) |  |  | 5 (7.9) | 58 (92.1) |  |
| Yes | 206 (91.6) | 19 (8.4) |  |  | 2 (5.3) | 36 (94.7) |  |

DSS, dengue shock syndrome.

^*^ Values are presented as n (%).
