# Supplementary material for: Demographic characteristics, clinical symptoms, biochemical markers and probability of occurrence of severe dengue: A multicenter hospital-based study in Bangladesh
Source: PLoS Negl Trop Dis. 2023 Mar 15;17(3):e0011161. doi: 10.1371/journal.pntd.0011161 (PMC10042364; doi:10.1371/journal.pntd.0011161)
Supplement: S7 Table — Abbreviation: WBC, white blood cell; ALT, alanine transaminase; AST, aspartate transaminase. (DOCX) [file pntd.0011161.s014.docx]

**S7 Table**. **The random forest model identified the important predictors on the prediction of severe dengue.**

|  | Mean Decrease Accuracy | *P* value |
| --- | --- | --- |
| Age | 23.59 | 0.01 |
| Education | 11.54 | 0.01 |
| Plasma leakage | 9.88 | 0.01 |
| Platelet count | 8.45 | 0.01 |
| Dyspnoea | 7.21 | 0.01 |
| WBC | 5.14 | 0.17 |
| Monthly income | 5.05 | 0.07 |
| Muscle pain | 4.81 | 0.10 |
| Haemoglobin | 4.52 | 0.18 |
| ALT | 4.20 | 0.58 |
| Itchiness | 3.84 | 0.03 |
| Hemorrhage | 2.41 | 0.15 |
| AST | 2.35 | 0.80 |
| Decreased appetite | 2.07 | 0.22 |
| Type of residence | 2.01 | 0.29 |
| Backpain | 1.70 | 0.50 |
| Duration of fever | 1.50 | 0.31 |
| Cough | 1.41 | 0.35 |
| Comorbidity | 0.94 | 0.58 |
| Headache | 0.89 | 0.46 |
| Dehydration | 0.73 | 0.41 |
| Rash | 0.59 | 0.51 |
| Sex | 0.40 | 0.55 |
| Joint pain | 0.38 | 0.60 |
| Lethargy | 0.30 | 0.55 |
| Abdominal pain | -0.47 | 0.64 |
| Vomiting | -0.94 | 0.79 |
| History of dengue | -2.32 | 0.92 |

WBC, white blood cell; ALT, alanine transaminase; AST, aspartate transaminase.
