# Supplementary material for: Demographic characteristics, clinical symptoms, biochemical markers and probability of occurrence of severe dengue: A multicenter hospital-based study in Bangladesh
Source: PLoS Negl Trop Dis. 2023 Mar 15;17(3):e0011161. doi: 10.1371/journal.pntd.0011161 (PMC10042364; doi:10.1371/journal.pntd.0011161)
Supplement: Attachment — Submitted filename: Review_comments.docx [file pntd.0011161.s015.docx]

This study will have strong public health and medical implications in terms of improving the care for dengue patients. Congratulations to the team. However, there are so many areas that require further improvement.

A definition on how severe dengue and non-severe dengue was not included in the method section. Please incorporate this.

There is no mention of sample size calculation in the method section. Please insert the method used for sample size calculation.

Line 93-95: Check the sentence structure

Line 106: replace “serological” with “serologically”

Line 110-111: Need a reason why you include patients during the convalescent phase of dengue infection

Line 118: “This study is reported per” should be replaced with “This study is reported as per”

Line 127-128: The variable “history of dengue” is confusing because you also included chikungunya as one of the categories. Suggested to revise the variable name (like the history of infection) or exclude chikungunya from the category

Line 137: There is no mention of how the collinearity was assessed in the method section. The inclusion of highly collinear variables in the model can produce a biased result.

Line 164: Suggested to begin a sentence with a text

Table 1: If data is available, it would be informative if you could also provide the type of co-morbidity because the severity of dengue differs according to the different diseases

Table 1: Information on how you managed the missing values would be helpful, particularly in the model. This information might better suit the method section.

Line 187: Is it possible to specify the type of haemorrhage and plasma leakage? Hemorrage could be melena, nose or GI bleeding, while plasma leakage may be in the form of pleural effusion, ascites….

Table 1 and Table 2: Non-severe dengue and severe dengue might better fit the column heading instead of non-severe and severe.

Table 1 and Table 2: There are inconsistencies in the reporting of P values. In table 1, you report three decimal places, while in table 2, you use only two decimal places.

Discussion

There is a strong need to improve the discussion section. In particular, the flow of information seems bit distorted. Some suggestions are:

Line 242-243: Severity in children can be mostly related to the fragile capillaries as compared to the adult population. This should be reported here.

Line 245-246: This sentence is fragmented.

Line 257-258: this sentence should be part of the result section

Line 260-262: Not clear. Will you break down the sentence to fewer lines for clarity

Line 256-267: Need revision of this sentence

Line 270-281: The topic sentence for this paragraph has no relevance with its content. Suggested to change the topic sentence.

Line 271-274: Provide reasons why identifying plasma leakage is challenging as compared to severe dengue.

Lilne 274-275: Is this the finding of your study or is it based on the past study? Suggested to relook at this structure.

You have not discussed dyspnea in the discussion section. This variable is found significant in the result.

It would be informative if you provide the summary of the findings and implications at the end of the discussion.
