# Supplementary material for: Demographic characteristics, clinical symptoms, biochemical markers and probability of occurrence of severe dengue: A multicenter hospital-based study in Bangladesh
Source: PLoS Negl Trop Dis. 2023 Mar 15;17(3):e0011161. doi: 10.1371/journal.pntd.0011161 (PMC10042364; doi:10.1371/journal.pntd.0011161)
Supplement: Attachment — Submitted filename: R1_ResponseLetter_dengue_PLOSNTD_21 Dec 2022.docx [file pntd.0011161.s016.docx]

**Response to Reviewers/Editor Comments**

# **Editor**

*Editor comments: The 3 reviewers found the study has potential but all of them raised major issues regarding both the analyses and the writing. We are happy to consider a resubmission of your manuscript if you think you will be able to address all the reviewers' comments.*

**Responses:** Thank you very much for your great support. We are grateful for the opportunity to revise our manuscript. We have addressed these comments carefully and have revised the manuscript as you and the reviewers suggested.

# **Reviewer #1**

1. *This study will have strong public health and medical implications in terms of improving the care for dengue patients. Congratulations to the team. However, there are so many areas that require further improvement.*

**Responses:** We are very thankful for your positive and constructive comments and suggestions, which are very helpful to our research.

2. *A definition on how severe dengue and non-severe dengue was not included in the method section. Please incorporate this.*

**Responses:** Thanks for your suggestions. We have added the definition of severe dengue and non-severe dengue in the Materials and Methods in the revised manuscript.

**Revisions:** “As per the National Guideline for Clinical Management of Dengue, symptomatic dengue cases were initially divided into three groups: dengue fever (group A), dengue fever with warning signs (group B), and severe dengue (group C). In this study, group A and group B were treated as the non-severe dengue group, while group C was treated as the severe dengue group [17]”. (**Lines 145-150**)

4. *There is no mention of sample size calculation in the method section. Please insert the method used for sample size calculation.*

**Responses:** Thanks for the reviewer’s comments. Essentially, this was a cross-sectional study and dengue cases were enrolled through convenience sample technique over a specific time frame. Therefore calculating sample size may not be statistically sound. We have revised the manuscript as follows:

**Revisions: “**This study was conducted in the Dhaka megacity (population= ~20M) of Bangladesh where a massive dengue outbreak occurred in 2019. We used convenience sampling technique to enroll hospitalized dengue cases from eight hospitals (five government hospitals and three private hospitals) between 15 August 2019 and 30 September 2019 [9] (**Lines 107-114**)

6. *Line 110-111: Need a reason why you include patients during the convalescent phase of dengue infection*

**Responses:** We used the convalescent phase of dengue infection for data collection so that we could record all the clinical and laboratory complications of the patients developed during their hospital stay. In addition, the recall bias should be negligible as most of the dengue patients reach convalescent within 7-10 days of starting symptoms. We updated the text accordingly.

**Revisions:** “Data were collected at a one-time point at the convalescent phase to record all the clinical and laboratory complications patients developed during their hospital stay.” (**Line 117-119**).

8. *Line 137: There is no mention of how the collinearity was assessed in the method section. The inclusion of highly collinear variables in the model can produce a biased result.*

**Responses:** Thanks for your consideration. We fully realized the collinearity issue in this study. We used classification and regression tree (CART) and random forest (RF) models which are nonparametric statistical methods that do not rely on assumptions about data distribution to handle multiple independent variables interacting. Moreover, we used the variance inflation factor (VIF) to estimate collinearity in the multiple logistic regression models. All VIF values were under 10, implying no substantial issue in collinearity. We have added the details of how we deal with multicollinearity in the revised manuscript.

**Revisions:** Method: “We used the variance inflation factor (VIF) to estimate multicollinearity in the multiple logistic regression models.” (**Lines 161-162**)

Results: “. All VIF values were under 10 in the multiple logistic regression model, implying no substantial issue in multicollinearity (S4 Table).” (**Lines 205-207**)

10. *Table 1: If data is available, it would be informative if you could also provide the type of co-morbidity because the severity of dengue differs according to the different diseases*

**Responses:** Thanks for your constructive suggestions. Unfortunately, such detailed data are unavailable in the research. We have added more discussion for the comorbidity in the discussion section.

**Revisions:** “However, the degree of severity of dengue may differ from the different diseases [23], the type of comorbidity should be investigated in the future study.” (**Lines 279-281**)

11. *Table 1: Information on how you managed the missing values would be helpful, particularly in the model. This information might better suit the method section.*

**Responses:** Thanks for your constructive comments. In this study, the missing values were less than 5% among most of the covariates. As you suggested we have added more details on how we deal with the missing data in the method section.

**Revisions:** “In this study, the missing values were less than 5% among most of the covariates. We used a listwise deletion method to deal with the missing data [18].” (**Lines 157-159**)

12. *Line 187: Is it possible to specify the type of haemorrhage and plasma leakage? Hemorrage could be melena, nose or GI bleeding, while plasma leakage may be in the form of pleural effusion, ascites….*

**Responses:** Sure. As per the reviewer request, we have analyzed and presented the data collected on hemorrhage and plasma leakage in the supplemental file **S7 Table**. We have revised and discussed in the revised manuscript.

**Revisions:** “The hemorrhage mainly occurred in the gum, hematochezia, and menorrhagia, and plasma leakage mainly in abdomen and chest (S5 Table).” (**Lines 209-211**)

“Furthermore, we found that plasma leakage occurred mainly in the abdomen and chest, and that people with ascites and pleural effusion were more likely to develop severe dengue.” (**Lines 304-306**)

13. *Table 1 and Table 2: Non-severe dengue and severe dengue might better fit the column heading instead of non-severe and severe.*

**Responses:** Thanks. Done

14. *Table 1 and Table 2: There are inconsistencies in the reporting of P values. In table 1, you report three decimal places, while in table 2, you use only two decimal places.*

**Responses:** Thank you for your suggestions. Two decimal places have been used in both tables 1 and 2.

15. *Line 242-243: Severity in children can be mostly related to the fragile capillaries as compared to the adult population. This should be reported here.*

**Responses:** Thanks for your insightful comments. We have added great point in this discussion section.

**Revisions:** “A piece of evidence from the Global Burden of Disease Study 2019 showed that the age-standardized mortality rate and disability-adjusted life years for dengue in children were higher than in older people [4], which might relate to fragile capillaries in children than adults [21].” (**Lines 266-271**)

16. *Line 270-281: The topic sentence for this paragraph has no relevance with its content. Suggested to change the topic sentence.*

**Responses:** We have changed the topic sentence accordingly. Thanks.

**Revisions:** “Furthermore, clinicians should be vigilant in identifying dengue patients, as prompt identification and early treatment of dengue remain the cornerstone of reducing morbidity and mortality [37].” (**Lines 342-344**)

17. *Line 271-274: Provide reasons why identifying plasma leakage is challenging as compared to severe dengue.*

**Responses:** Thanks for your great comment. We have supplemented the discussion about plasma leakage in the Discussion.

**Revisions:** “Plasma leakage is an important outcome for dengue, as most complications occurred in this group. Identifying all plasma leakage is an important factor in preventing complications, but neither WHO classification fully covers the plasma leakage subgroup [29, 30]. In addition, the definition and development of criteria for diagnosing plasma leakage have long been neglected, resulting in challenging and underreporting of plasma leakage. It is recommended that standardization of diagnosis and reporting of plasma leakage should be a research priority for dengue [31].” (**Lines 306-312**)

18. *You have not discussed dyspnea in the discussion section. This variable is found significant in the result.*

**Responses:** Thanks. We have supplemented the discussion about dyspnoea in the revised manuscript.

**Revisions:** “Furthermore, we found that dyspnoea was a vital sign of severe dengue. Published research showed a significant association of dyspnoea with shock, which may result from fluid overload due to pulmonary congestion caused by capillary leakage [36]. Early recognition and treatment of dyspnea are essential for preventing severe dengue.” (**Lines 326-341**)

19. *It would be informative if you provide the summary of the findings and implications at the end of the discussion.*

**Responses:** Thanks for your comments. We have added the conclusion at the end of the discussion.

**Revisions:** “In conclusion, combined with socio-environmental factors, early recognition and treatment of dyspnoea, plasma leakage, and hemorrhage are vital to the prevention of severe dengue. The research provides new evidence to identify key risk factors that contribute to severe dengue cases, which would add new evidence for the early identification and treatment of severe dengue fever. Future research is required to further explore the progression and shifting from non-severe dengue to severe dengue based on the research finding.” (**Lines 379-385**)

21. *Line 93-95: Check the sentence structure*

*Line 106: replace “serological” with “serologically”*

*Line 118: “This study is reported per” should be replaced with “This study is reported as per”*

*Line 127-128: The variable “history of dengue” is confusing because you also included chikungunya as one of the categories. Suggested to revise the variable name (like the history of infection) or exclude chikungunya from the category*

*Line 164: Suggested to begin a sentence with a text*

*Line 245-246: This sentence is fragmented.*

*Line 257-258: this sentence should be part of the result section*

*Line 260-262: Not clear. Will you break down the sentence to fewer lines for clarity*

*Line 256-267: Need revision of this sentence*

*Line 274-275: Is this the finding of your study or is it based on the past study? Suggested to relook at this structure.*

**Responses:** Thanks for your comments. We have modified all the above according to your suggestion, and a number of grammatical errors have been corrected.

# **Reviewer #2**

1. *This is an exploratory analysis, so there is no specific hypothesis.*

*This is a large population from Bangladesh including both severe and non-severe dengue cases, which is appropriate for exploring variables that may differentiate between severe and non-severe dengue.*

**Responses:** Thank you very much for the positive and insightful comments on our manuscript.

2. *Can the authors please state how dengue was diagnosed? Did they use PCR, serology, both? Do they have data on which serotypes were causing infection?*

**Responses:** Thanks for the suggestion. Dengue diagnosis was serology based in this study as PCR-based diagnostic test is unavailable for clinical use in Bangladesh. We have updated the text on this **(Lines 114-116**). Unfortunately, we do not have data on serotypes, which was discussed as a limitation.

**Revisions:** “The diagnosis was confirmed serologically by the positivity of antibodies against the dengue nonstructural glycoprotein 1 (NS1).” (**Lines 114-116**)

“Lastly, given that the risk of developing severe dengue could be associated with secondary infections [42]. The study did not explore this relationship because of lack of detailed data on IgG (an indicator of secondary infection) and dengue virus serotypes.” (**Lines 375-378**)

3. *Since this is a cross-sectional study, I expect only 1 time point was measured. However, the author's should state this explicitly. If only one time point was measured, I think the authors should report and control for days post symptom onset in their models. Per the data form shared in the supplementary material, it looks like symptom onset day may have been collected.*

**Responses:** Yes. We collected data at a one-time point at the convalescent phase so that we could record all the clinical and laboratory complications the patients developed during their hospital stay. As per your suggestion, we updated the text accordingly.

**Revisions:** “Data were collected at a one-time point at the convalescent phase to record all the clinical and laboratory complications patients developed during their hospital stay.” (**Lines 117-119**)

4. *In the methods, they explain that they dichotomized the data as non-severe and severe dengue, and then they cite the WHO criteria. This is confusing to me because, according to their models, the three variables with the strongest association with severe dengue (plasma leakage, hemorrhage, and dyspnea) are all part of the WHO criteria for severe dengue. If they used the WHO criteria to define severe dengue wouldn't it follow that the models would reflect the WHO criteria when identifying variables associated with severe dengue? Maybe I am misunderstanding how these models work, but then I think it would be helpful to walk the reader through this a bit more.*

**Responses:** As you mentioned, the WHO has defined severe dengue by one or more of the following: (i) plasma leakage that may lead to shock (dengue shock) and/or fluid accumulation, with or without respiratory distress, and/or (ii) severe bleeding, and/or (iii) severe organ impairment based on the WHO criteria. 2009 WHO classification categories do not have a good overlap with the plasma leakage or hemorrhage subgroup, only severe plasma leakage or severe bleeding could be defined as severe dengue. In this study, only 38.0% dyspnoea, 33.5% plasma leakage, and 35.4% haemorrhage were belonging to severe dengue (S1 Table), which meant that not all severe dengue had these symptoms. Our research suggested that further research is required to assess the degree of plasma leakage, haemorrhage and dyspnea in clinical practice for the severe dengue.

5. *Can the authors do a bit better job explaining what their study adds to the literature? For example, in line 238 - 239, they state that "The predictive probability of occurrence of severe dengue achieved 92.9% among people aged ≤ 12.5 years with dyspnoea and plasma leakage." But, if a clinician just follows the WHO criteria, then 100% of patients with plasma leakage would meet the criteria for severe dengue. The same is true for line 257, where they report that those with hemorrhage were 2.33 times more likely to develop severe dengue. But, if a clinician just follows WHO criteria, then 100% of patients with dengue and hemorrhage have severe dengue. So, I just don't really understand what the added value is of the model. Could the authors help readers understand that a bit better?*

**Responses:** Thanks for your comments. This is a very good point. Our model aims to identify the combined effect of risk factors on all severe dengue cases, which include cases with key symptoms such as hemorrhage and plasma leakage etc, and without these key symptoms. In the research, however, 33.5% of severe dengue cases don’t have the key symptoms. Our results will provide very useful warning signals for all dengue case for clinical doctors. We also have added the discussion in the revised manuscript.

**Revisions:** “In the research, we used machine learning algorithms, including CART and RF, to find key risk factors and to identify the combined effect of risk factors on all severe dengue cases, which include cases with key symptoms such as hemorrhage and plasma leakage etc, and without these key symptoms. Our results will provide very useful warning signals for all dengue cases for clinical doctors.” (**Lines 354-359**)

“The research provides new evidence to identify key risk factors that contribute to severe dengue cases, which would add new evidence for the early identification and treatment of severe dengue fever.” (**Lines 380-383**)

6. *The authors make a few conclusions stating that their data provides "new evidence" (line 292) or "enhanced insight into clinical treatment" (line 294), but I am not sure that these conclusions are well justified. Can the authors explain what the new evidence is and how this will impact clinical treatment and prevention? It seemed that the clinical markers they report here are all consistent with the WHO criteria, so they seem to be confirming the WHO criteria rather then adding to them. The age and educational associations with severe dengue could potentially be helpful though. For example, educational public health campaigns might target lower income areas and families with children.*

**Responses:** Thank you very much for your positive and constructive suggestions and comments. According to the WHO, coordinated processes for early detection, classifying, treating, and referring severe dengue lower mortality to less than 1%. Finding biomarkers that can reliably predict the development of severe dengue in symptomatic individuals is one of the main focuses of current research efforts. During the transition from the febrile period to the critical period, WHO highlights several clinical warning signs as potential signs of impending exacerbation. Therefore, establishing an early warning system for severe dengue based on these factors is essential for triaging in endemic areas and optimizing resource utilization. However, the diagnostic criteria of WHO have not provided detailed guidelines of quantified combination effects. Clinic doctors may focus on clinic symptoms but ignore other risk factors. The research will add new evidence to identify key risk factors among socio-environmental factors and biomarkers in all dengue cases. We also have added the discussion in the revised manuscript.

**Revisions:** “The research provides new evidence to identify key risk factors that contribute to severe dengue cases, which would add new evidence for the early identification and treatment of severe dengue fever.” (**Lines 380-383**)

“Therefore, educational public health campaigns might target lower-income areas, individuals with comorbidities, and families with children.” (**Lines 288-289**)

7. *This is just a suggestion, but it might actually be informative to do an unsupervised clustering analysis where they just see which variables cluster together without considering whether the dengue is severe or not. This would remove the WHO definitions from the model and might allow the authors to just observe which variables cluster together and potentially independently confirm the WHO definitions.*

**Responses:** Thanks for your suggestion. As you mentioned, the unsupervised clustering analysis just sees which variables cluster together without considering whether the dengue is severe or not. Unfortunately, we do not know how these clustered variables are related to severe dengue fever, nor can they play a predictive role in severe dengue. In this study, we used the random forest (RF) model to find the key risk factors of severe dengue by machine learning and used classification and regression tree (CART) to identify the combined effect of risk factors on all severe dengue cases. Unsupervised clustering analysis is often used in exploratory research. RF and CART are more robust and reliable than unsupervised clustering analyses.

8. *I think the associations between severe dengue and age and education levels are interesting and notable, but of course may not be generalizable to all cohorts. This limited generalizability should be noted.*

**Responses:** Thanks for your comments. As with all research, it should be careful when the results are applied to other regions due to regional differences. Therefore, we add this limitation to the revised manuscript.

**Revisions:** “Third, due to regional differences, the results of this study may not be generalizable to other regions.” (**Lines 371-372**)

9. *The analysis does match the analysis plan and the tables are clear, well structured, and helpful.*

**Responses:** Thanks for your positive comments.

10. *The figures are a bit blurry, and the explanation of figure 1 seems to have a typo (line 220).*

**Responses:** We have updated all the figures and corrected the typo accordingly.

11. *In line 283, the authors state that "This study is the first to study severe dengue from a multi-index and multimethod perspective." Again, I am not sure this is quite true. There are a number of meta-analyses and cohort studies that have examined severe dengue and the authors may consider citing some of these. For example, Yaun et al. PLOS NTD, 2022. Sangkaew et al. Lancet ID, 2021. Paz-Bailey et al. JID 2022.*

**Responses:** Thanks for your comments. As you mentioned, there are many studies exploring the factors of severe dengue, but few studies identify the combined effect of risk factors and explore which are more important among so many factors for severe dengue. Combining different characteristics to predict severe dengue fever and prevent dengue fever from developing into a severe disease is very helpful for reducing the burden on individuals, families, or society, especially in low-income regions. This is what distinguishes this study from other studies, and it is also the question that this study aims to address. As mentioned in comment 6, the research will add new evidence to identify key risk factors among socio-environmental factors and biomarkers in all dengue cases. We also have added the discussion in the revised manuscript.

**Revisions:** “In the research, we used machine learning algorithms, including CART and RF, to find key risk factors and to identify the combined effect of risk factors on all severe dengue cases, which include cases with key symptoms such as hemorrhage and plasma leakage etc, and without these key symptoms. Our results will provide very useful warning signals for all dengue cases for clinical doctors.” (**Lines 354-359**)

“The research provides new evidence to identify key risk factors that contribute to severe dengue cases, which would add new evidence for the early identification and treatment of severe dengue fever.” (**Lines 380-383**)

12. *Line 293: seems to have a typo of "n"*

**Responses:** We have corrected the typo. Thanks.

13. *Line 253: what do they mean by nursing? Is there some data to suggest that breastfeeding women are at higher risk for severe dengue? Do they know if many of the women in their cohort were breastfeeding? Since they have the data, it might be helpful to see if the sex differences were true in children and adults. If they only saw sex differences in adults, then potentially there is some hormonal component?*

**Responses:** In this study, we found the prevalence of severe dengue was 50.7% for women, and 38.7% for men among participants aged between 1.9 and 11.5 years. A preponderance of females in severe dengue cases has been reported in previous studies which explained that it may be related to the time of presentation or differences in the type of care provided to girls and boys [1,2]. What we want to express here is the sex-specific may be related to differences in the type of care. We have corrected it accordingly in the revised manuscript (**Line 271**).

It’s a good idea to explore if breastfeeding women are at higher risk for severe dengue. Unfortunately, we did not collect breastfeeding-related data, which limited our study. But you really provide great ideas for our future research. Thank you very much.

Reference:

[1] [Epidemiological Factors Associated with Dengue Shock Syndrome and Mortality in Hospitalized Dengue Patients in Ho Chi Minh City, Vietnam in: The American Journal of Tropical Medicine and Hygiene Volume 84 Issue 1 (2011) (ajtmh.org)](https://www.ajtmh.org/view/journals/tpmd/84/1/article-p127.xml)

[2] [Dengue haemorrhagic fever in children in the 1996 Delhi epidemic - PubMed (nih.gov)](https://pubmed.ncbi.nlm.nih.gov/10492762/)

# **Reviewer #3:**

1. *This is a large and unique clinical cohort which includes 1,090 participants (932 non-severe patients and 158 severe patients). This study also includes both adult and pediatric participants which allows them to evaluate age-dependent differences in the development of severe dengue.*

*The authors use multiple non-parametric measures to identify patterns in their data which have enabled them to quantify the clinical, demographic and laboratory parameters which increase the probability of developing severe dengue.*

*Patient questionnaires were performed during the convalescent phase of dengue infection and therefore may not be sensitive or specific for the prognosis of severe dengue.*

*This is a single cohort study and it is unclear if these findings could be extrapolated to other dengue endemic areas which may have different epidemiological aspects or common co-infections such as malaria.*

**Responses:** Thank you very much for your positive comments. As with all research, it should be careful when the results are applied to other regions due to regional differences. Therefore, we add this limitation to the revised manuscript. However, as reviewer 1 and reviewer 2 mentioned, the results could have strong public health and medical implications in terms of improving the care for dengue patients and providing an important reference for dengue education and early identification and treatment of severe dengue by clinicians.

**Revisions:** “Third, due to regional differences, the results of this study may not be generalizable to other regions.” (**Lines 371-372**)

2. *The presentation of the results from Figure 1 describing the CART model are difficult to interpret. Generating a figure which more clearly visualizes the mean and SD for each node would improve understanding of the results.*

**Responses:** We have updated all the figures. Thanks for your suggestions.

3. *The authors do not fully describe the limitations of using study questionnaires during the convalescent phase of infection or discuss potential recall biases during interviews. Given that there was no association between primary and secondary dengue infections and severity, a well known risk factor for developing severe dengue, it would be important to discuss these challenges.*

**Responses:** This is a valuable suggestion. Our study did not explore the relationship of primary and secondary infections in to severity of the disease. For your info, we did not have enough serological data for establishing secondary infection (assessing of IgG). We have discussed this in the revised manuscript as a limitation

**Revisions: “**Lastly, given that the risk of developing severe dengue could be associated with secondary infections [42]. The study did not explore this relationship because of lack of detailed data on IgG (an indicator of secondary infection) and dengue virus serotypes.” (**Line 375-378**)

4. *The quality of the tables and figures should be improved. Figure 1 and 2 are blurry and at times the wording is not clear.*

**Responses:** We have updated all the figures. Thanks for your suggestions.

5. *The findings of this study generally coincide with the known associations of severe dengue which are part of the WHO classifications (thrombocytopenia, hemorrhage, plasma leakage). Children have also previously been found to be at increased risk for developing severe dengue and to have higher mortality rates. Therefore there is limited novelty in these findings and it is unclear what additional benefit they would add to support clinical decision making.*

**Responses:** Thank you very much for your comments. According to the WHO, coordinated processes for early detection, classifying, treating, and referring severe dengue lower mortality to less than 1%. Finding biomarkers that can reliably predict the development of severe dengue in symptomatic individuals is one of the main focuses of current research efforts. During the transition from the febrile period to the critical period, WHO highlights several clinical warning signs as potential signs of impending exacerbation. Therefore, establishing an early warning system for severe dengue based on these factors is essential for triaging in endemic areas and optimizing resource utilization. However, the diagnostic criteria of WHO have not provided quantified combination effects. Clinic doctors may focus on clinic symptoms but ignore other risk factors. In the research, we used machine learning algorithms, including classification and regression tree (CART) and random forest (RF) models, to find the key risk factors and identify the combined effect of risk factors on all severe dengue cases. The research will add new evidence to identify key risk factors among socio-environmental factors and biomarkers in all dengue cases. We also have added the discussion in the revised manuscript.

**Revisions:** “In the research, we used machine learning algorithms, including CART and RF, to find key risk factors and to identify the combined effect of risk factors on all severe dengue cases, which include cases with key symptoms such as hemorrhage and plasma leakage etc, and without these key symptoms. Our results will provide very useful warning signals for all dengue cases for clinical doctors.” (**Lines 354-359**)

“The research provides new evidence to identify key risk factors that contribute to severe dengue cases, which would add new evidence for the early identification and treatment of severe dengue fever.” (**Lines 380-383**)

6. *Given the strong correlation with age with disease severity, the authors should consider subsampling their cohort by age and evaluating whether there are specific associations with severe dengue in adults only and children only. This may aid in generating more precision approaches to predicting severe dengue.*

**Responses:** Thanks for your insightful suggestions. We divided the study participants into two groups (i. e. >12.5 years and <12.5 years) by the cut point from the CART in Fig 1. We also found that age was a more important factor in participants aged <12.5 years (7^th^) and participants aged >12.5 years (5^th^). More discussion on age and disease severity has been added in the discussion section.

**Revisions:** “Our research found that age was associated with the risk of severe dengue. A piece of evidence from the Global Burden of Disease Study 2019 showed that the age-standardized mortality rate and disability-adjusted life years for dengue in children were higher than in older people [4], which might relate to fragile capillaries in children than adults [21]. As previous studies reported, we found that the prevalence of severe dengue increased in specific populations, such as the older with comorbidities [22].” (**Lines 266-275**)

 “Therefore, educational public health campaigns might target lower-income areas, individuals with comorbidities, and families with children. (**Lines 288-289**)

7. *The authors used the 2009 WHO criteria to classify patients. These criteria include uncomplicated dengue, dengue with warning signs (an intermediate classification which has higher likelihood of progressing to severe dengue) and severe dengue. If the authors have the data evaluating the clinical and laboratory findings in uncomplicated dengue patients and dengue with warning signs patients at admission who then progressed to severe dengue during their hospitalization would add to predictive power of their findings.*

**Responses:** Thanks for your constructive comments. It’s a really interesting idea. Unfortunately, we don’t have such detailed hospitalization data to assess the progress from uncomplicated dengue to severe dengue in the research. We have added these suggestions for future research perspective in discussion section.

**Revisions:** “Future research is required to further explore the progression and shifting from non-severe dengue to severe dengue based on the research finding.” (**Lines 383-335**)

8. *Since the authors used the 2009 criteria to diagnose severe dengue which include organ impairment as part of the criteria it’s unclear how these patients may have affected outcomes as patients with plasma leakage/hemorrhage and organ damage may have two distinct immuno-pathogeneses. Separating the severe dengue patients by syndrome may further clarify the association of plasma leakage and hemorrhage with severity.*

**Responses:** Thanks for your comments. We divided the participants into the DSS group (dengue shock syndrome) and the non-DSS group. Our research showed that severe dengue in the non-DSS group was significantly higher in participants with plasma leakage/hemorrhag than in those without plasma leakage/hemorrhag. The results of this study are more valuable for early detection and diagnosis of severe dengue in people without DSS (see S6 Table). We have revised and discussed in the revised manuscript.

**Revisions:** “Furthermore, severe dengue in the non-DSS group was significantly higher in participants with plasma leakage or hemorrhage than in those without plasma leakage or hemorrhage (S6 Table).” (**Lines 211-213**)

“The results of our study identified possible combinations of severe dengue, which would provide an essential reference for the early identification, and treatment of severe dengue fever, especially for patients without DSS.” (**Lines 351-353**)
